# Boylston Prize Essay, 1871

**Published:** 1872-03

**Authors:** 


*Boylston Prize Essay*, 1871. *Diseases of the Skin; the recent advances in their Pathology and Treatment.* By B. JOY JEFFRIES, A. M., M. D. Boston : Alex. Moore, 1871.

Dr. Jeffries has in an original and intelligent manner, placed before us a very interesting statement of the recent advances in the Pathology and Treatment of Skin Diseases. After reading his essay, we come to the conclusion that he is possessed of that rare gift of saying many things in a few words. His treatment of the subject is brief, original and to the point. In speaking of the vexed question of classification, he says: "A teacher may, most usefully for the student, devote, hours to a thorough and practical description of the symptoms and treatment of the various phases of eczema, without once alluding to where it, as a whole, or its several appearances, belong in any one's classification. Some general order of bringing cutaneous diseases before his class is, of course, necessary for a teacher; but the less the student hears of the special order or classification, and the more of the particular disease, the more successful will be the treatment of his cases, and the better will he be enabled to classify for himself, whilst at the same time he has become trained in observation."

It must not be understood that Dr. Jeffries wishes to throw aside all classification; but that he wishes the pathology and treatment of disease to be of the first, and the classification of secondary consideration. The book is full of valuable intelligence and should not fail to be in the hands of those of the profession who wish to live up to the times.

